# Role of Peptide Hormones in the Adaptation to Altered Dietary Protein Intake

**DOI:** 10.3390/nu11091990

**Published:** 2019-08-23

**Authors:** Adam J. Rose

**Affiliations:** Nutrient Metabolism & Signalling Laboratory, Department of Biochemistry and Molecular Biology, Metabolism, Diabetes and Obesity Program, Biomedicine Discovery Institute, Monash University, Clayton 3800, Australia; adam.rose@monash.edu

**Keywords:** glucagon, FGF21, PYY, insulin, endocrine, amino acid

## Abstract

Dietary protein profoundly influences organismal traits ultimately affecting healthspan. While intracellular signalling downstream of altered amino acid supply is undoubtedly important, peptide hormones have emerged as critical factors determining systemic responses to variations in protein intake. Here the regulation and role of certain peptides hormones in such responses to altered dietary protein intake is reviewed.

## 1. Introduction

Introduction of the major macronutrients, dietary protein is a powerful determinant of longevity, health span, and reproduction through both behavioural changes such as food intake as well as whole-body and cellular metabolism [1]. This is likely fundamentally related to the fact that amino acids, the building blocks of protein, are an essential dietary component [2] and that certain amino acids are critical for vital processes such as gut function, immune response and neurotransmitter synthesis [3]. While there are clear intracellular signalling nodes such as mTORC1 [4] and GCN2 [5] as well as possible cell surface receptors for amino acids [6], it is becoming clear that hormones, in particular peptide hormones, are critical components relaying the systemic homeostatic responses to variations in dietary protein intake. Although amine (e.g., tryptophan derived serotonin [7]) and steroid hormones are likely to be important as well, here we review the regulation and role of certain peptide hormones in the physiological responses to altered dietary protein. Rather than being exhaustive, this review is intended to be easily *digestible*, and summarises the major peptides hormones from the gut, pancreas, liver, and adipose tissue, and the information available about their responsiveness, and physiological role in variations, to dietary protein intake.

## 2. Peptide Hormones from the Alimentary Tract

### 2.1. Stomach Derived Peptide Hormones

#### 2.1.1. Gastrin

Gastrin is involved in the acute regulation of protein digestion [8]. In addition to other stimuli, gastrin is mainly secreted from the G cells of the gastric antrum and duodenum via vagal stimulation as well as gastrin-releasing peptide, secondary to ingestion of peptides [8]. Following secretion into the bloodstream, gastrin then travels to the gastric fundus and acts on parietal cells to secrete hydrochloric acid which alters gastric pH which cleaves pepsinogen into pepsin to aid in peptide chemical digestion [8].

#### 2.1.2. Ghrelin

Ghrelin is secreted by the stomach, mainly by the gastric fundus in response to lack of food [9]. The levels of ghrelin typically rise shortly before and fall shortly after food consumption, and is thought to have a role in meal hunger and food intake initiation (i.e., feeding) via brain orexigenic circuits [10]. Although this is the well-known role of ghrelin, the effects are pleiotropic including broader effects on mood, energy balance, as well as gastric-, cardiovascular-, and muscular-function [10]. The meal response to suppress ghrelin depends on total caloric supply but does not seem to be selective for a particular macronutrient, although carbohydrate appears to be dominant [11].There is variable information on whether ghrelin is secreted in response to dietary protein alone [11]. Nevertheless, a well-controlled study concluded that the satiating effects of increased dietary protein does not relate to alterations in ghrelin [12]. Whether ghrelin is involved in protein-specific hunger, as well as whether ghrelin is involved in many of the effects of dietary protein on organismal function, remains to be formally investigated.

### 2.2. Small/Large Intestine Derived Peptide Hormones

#### 2.2.1. Secretin

Secretin is the first discovered hormone by the seminal work of Bayliss and Starling [13]. It is secreted by the S-cells of the duodenum in response to meal feeding and affects multiple tissues by acting on secretin receptors and increasing cell growth and proliferation [13]. Secretin has a known role for affecting gastric acid secretion by the stomach and pancreatic secretory responses to meal feeding related acidification of the duodenum whereby it stimulates pancreatic fluid (ca. bicarbonate) release [13] to neutralise the duodenal acid load from the stomach. It also has a role in satiety [14], and it may mediate satiety by affecting postprandial thermogenesis via brown fat action [15], at least in mice. In terms of macronutrient effects, there is not much known, but one study showed no effect of a protein rich meal on postprandial blood secretin levels [16]. Whether secretin is involved in the acute and chronic effects of altered protein balance is yet to be systematically investigated.

#### 2.2.2. Cholecystokinin

Cholecystokinin (CCK) is secreted by the I-cells of the duodenal and jejunal mucosa [17] in response to a meal and acts as a satiety hormone, mainly by affecting meal size and duration [11,18]. It acts on CCK receptors within the stomach and duodenum, as well as vagal afferents within the gut to increasing firing to the hindbrain regions [11]. CCK receptors are also expressed within the hindbrain and hypothalamus and thus CCK can signal in multiple ways to affect satiation [19]. CCK is release in response to nutrients in the duodenal lumen, with fat and protein being more potent than carbohydrate [20]. Dietary fibre can also affect CCK secretion [20]. Concerning protein, digestion of protein is required for its effect on sustained CCK release [11] but whether CCK has a role in the regulation of somatic responses to acute and chronic changes in dietary protein is unknown.

#### 2.2.3. Glucagon-Like Peptide 1

Glucagon-like peptide 1 (GLP1) is derives from the proglucagon gene, and is secreted mainly by the L-cells of the distal small intestine and colon in response to food intake [21]. GLP1, mainly known for its incretin function where it enhances nutrient stimulation/inhibition of pancreatic hormone release [21], is also an anorexic hormone acting on the GLP1 receptor in multiple tissues including the gut, pancreas, brainstem, hypothalamus, and vagal-afferent nerves [22,23]. In response to food intake, the response of secretion is rapid [24]. This can occur via indirect neural mechanisms as well as direct effects on the intestinal L cells by nutrients [25]. In terms of sensitivity to different macronutrients, GLP1 responses are highest after a protein rich meal when compared with fat or carbohydrate rich meals [26,27]. Several lines of evidence suggest that certain amino acids such as glutamine and arginine appear to be important GLP1 secretagogues [28]. Whether GLP1 affects the organismal responses to altered dietary protein is yet to be properly investigated.

#### 2.2.4. Glucagon Like Peptide 2

Glucagon like peptide 2 (GLP2) also derives from the proglucagon gene, and is typically co-secreted with GLP1 [25]. GLP2 mainly has a known role in affecting intestinal mucosal epithelia proliferation/growth and is proposed to be a sensor for mucosal epithelium integrity to maintain nutrient absorption capacity [25]. A recent study has shown that GLP2 also affects intestinal amino acid transport directly [29]. Very little is known about how and which specific nutrients affect GLP2 secretion and whether GLP2 affects the systemic responses to changes in dietary protein. 

#### 2.2.5. Glucose-Dependent Insulinotropic Polypeptide

Glucose-dependent insulinotropic polypeptide (a.k.a. gastric inhibitory polypeptide; GIP) is released from intestinal K-cells predominantly in the duodenum in response to the presence of nutrients in the intestinal lumen [30]. GIP is mainly known to affect meal-induced glucose-stimulated pancreatic insulin secretion [31], GIP also affects postprandial adipose tissue lipid metabolism by adipose tissue action [32,33], but not gastric emptying [34]. GIP levels are mainly affected by carbohydrate and fat [35,36,37,38], but not by protein [37,38].

#### 2.2.6. Peptide Tyrosine-Tyrosine

Peptide tyrosine-tyrosine (PYY) is released from the intestinal L cells from the distal parts of the GI tract including the ileum and colon [39] in response to meal feeding [40]. PYY is involved in a wide range of postprandial functions, including the slowing of gastric emptying and digestive processes to improve nutrient absorption as well as affecting insulin secretion and glucose homeostasis [41,42]. The postprandial PYY secretion is biphasic, with initial stimulation by neural pathways from the foregut followed by intestinal lumen nutrient stimulation [11]. The rise and postprandial PYY levels are typically proportion to total food caloric intake but may also be affected by macronutrient content [21]. There is a lack of consensus about which particular macronutrients play a dominant role in stimulating PYY secretion [11]. However, a key study demonstrated that in humans, meals high in protein induced the greatest postprandial increase in PYY [43]. In addition, PYY is required for the satiating and weight-reducing effects of a high-protein diet in mice [43] probably via direct actions on the brain feeding circuits [44,45].

#### 2.2.7. Fibroblast Growth Factor 19

Fibroblast growth factor 19 (FGF19; FGF15 in rodents) is secreted from the enterocytes of the small intestine in response to meal feeding and coordinates postprandial nutrient homeostasis [46]. The main ascribed role of FGF19 is in bile acid homeostasis whereby it responds to bile acids and affects liver bile acid metabolism and gallbladder filling [47]. It also has other profound effects on liver carbohydrate, lipid, and protein metabolism [46]. Very little is known about how FGF19 is affected by different dietary macronutrients and whether FGF19 affects the response to alerted dietary protein.

## 3. Pancreatic Peptide Hormones

### 3.1. Insulin

Insulin is the most well studied postprandial hormone. It is secreted by the beta-cells of the pancreatic islets in response to meal feeding [11]. It acts on insulin responsive target tissues that express the insulin receptor such as muscle, adipose tissue and liver as well as the brain to coordinate proper glucose and energy homeostasis [48]. The beta cells respond to small monomeric metabolites such a monosaccharides, L-amino acids and ketone bodies [11,49], and these effects can be potentiated by other intestinal peptide hormones (discussed above). The most important of these is glucose, with beta cells being exquisitely sensitive to hepatic portal vein glucose concentrations [50]. In vivo [51,52,53,54] and ex vivo [49] studies have shown that amino acids can stimulate pancreatic insulin secretion with nearly amino acids having the capacity to stimulate insulin secretion [49]. However, since amino acids also stimulate glucagon secretion (see below) and increase endogenous glucose production, effects that can stimulate beta-cell insulin secretion [49], it remains unclear whether these effects are direct. In terms of regulating protein/amino acid homeostasis, insulin limits glucagon secretion [55] and affects amino acid uptake and protein synthesis [49,56]. Chronic dietary protein enrichment induces insulin resistance [57,58,59]. Chronic dietary protein restriction decreases insulin levels [58,60,61], but this is likely due to effects on heightened glucose metabolism by FGF21 (discussed below).

### 3.2. Glucagon

Glucagon (GCG) is typically known as a fasting hormone [62], but is robustly secreted by the pancreatic alpha-cells during a mixed meal containing all three major macronutrients [55]. Protein feeding and associated increased blood amino acids can stimulate glucagon secretion in vivo [53,54,63,64] and ex vivo [65,66], and are bone-fide glucagon secretagogues [67]. Nearly all amino acids can stimulate GCG secretion with the exception of the branched chain amino acids leucine, isoleucine, and valine [53]. Nonetheless, high prevailing glucose concentrations can supress this effect under normal circumstances [66], but this effect is appears to be abolished in frank type 2 diabetes [63,68]. Several recent studies using glucagon gene [69,70] or glucagon receptor gene [71,72,73,74,75] mutant mice as well as glucagon receptor antagonists [71,73,74,76], have established a firm role for glucagon in controlling systemic amino acid homeostasis via liver action. Importantly, this interrelationship seems to also exist in humans [77]. In addition, studies have revealed that either gain- [78] or loss- [79] of glucagon action can exert effects via the liver-derived hormone FGF21, but these effects may relate to dysregulated amino acid homeostasis (see above and below). Brain GCG action has been shown to convey improved glucose metabolism [80], but not hypophagia, with heightened dietary protein intake [81], which is controversial as high dietary protein intake is normally associated with worsened glucose homeostasis (see above).

### 3.3. Amylin

Amylin (a.k.a. amyloid polypeptide) is an anorexigenic hormone secreted by the pancreatic islet beta-cells [82] in response to meal feeding and reduces meal size and number [83,84] by mechanisms that are not fully understood. Although responsive to mixed meal feeding [85], this is probably mostly related to dietary carbohydrate supply [86,87].

### 3.4. Pancreatic Polypeptide

Pancreatic polypeptide (PP) is secreted by the F-cells of the pancreatic islets [88] in a biphasic manner in response to meal feeding PP affects energy balance by supressing food intake and gastric emptying [89,90]. PP secretion is stimulated by the parasympathetic nervous system and is largely unrelated to meal calories and macronutrient balance [11].

### 3.5. Somatostatin

Somatostatin (SST; a.k.a. growth hormone inhibiting hormone) is secreted by the delta-cells of the pancreas [91]. In general, SST inhibits neuroendocrine secretory process [91] and impacts gastric acid secretion [92] and pancreatic islet insulin and glucagon secretion [93]. Whether and how particular macronutrients affect SST, and the involvement of physiological responses during feeding, is poorly understood.

## 4. Liver-Derived Peptide Hormones

### 4.1. Fibroblast Growth Factor 21

Fibroblast growth factor 21 (FGF21), an hepatocyte/liver-derived peptide hormone, has emerged as a potent regulator of systemic response to altered nutrient supply [94]. Originally thought to be responsive to fasting and ketogenic diet feeding [95,96], in humans the most robust responses in FGF21 are in response to sucrose/carbohydrate feeding [97,98,99] or chronic protein restriction [60,100,101,102,103,104], effects which are paralleled in rodents [60,101,105,106,107,108,109]. When comparing all three major macronutrients simultaneously, dietary protein restriction emerges as the most potent variable affecting blood levels of FGF21 [60,108]. In response to dietary protein restriction, food intake is heightened along with energy expenditure, effects that require liver-derived FGF21 [60,61,101,103]. However, FGF21 does not mediate the effect of decreased dietary protein on the lowering of systemic protein oxidation and ureagenesis [60]. Liver FGF21 is also required for low-protein diet induced enhancement of age-related female ovarian follicle reserve [110], and thus may confer effects of dietary protein on age-related fertility. While FGF21 can signal though both liver, adipose tissue, and the brain [94], signalling in the brain is required for the feeding and metabolic responses to protein restriction [111]. While the biological basis for the increased FGF21-driven feeding during protein restriction is to increase protein intake [111], that of the increased energy expenditure remains elusive. Of note however, is that the increased energy expenditure during protein restriction can be uncoupled from increased feeding [112] and FGF21 confers dietary protein restriction mediated protection from obesity-related metabolic dysfunction [60,61,111,113]. Of protein, it is probably dietary amino acid restriction to the liver that confers the effect on FGF21 and related metabolic remodelling [60,102,113,114,115], and while the precise amino acids conferring these effects remain unclear, essential amino acids seem likely [114,116,117,118,119].

### 4.2. Insulin-Like Growth Factor 1

Insulin-like growth factor 1 (IGF1) is a liver derived hormone and part of the growth hormone-IGF axis. Whether altered dietary protein affects IGF1 is currently controversial with some studies showing a positive relationship [60,120,121], and other studies showing no relationship [109], with dietary protein intake and blood/hepatic IGF1 levels. Nonetheless, no studies have currently been undertaken to examine the role of liver-derived IGF1, and IGF1 signalling, in conferring the systemic metabolic effects of altered dietary protein.

## 5. Adipose Tissue Derived Peptide Hormones

### 5.1. Leptin

Leptin is an adipose tissue derived peptide hormone with levels correlating with whole-body fatness in multiple mammalian species. Studies of both human and mouse leptin receptor mutants have revealed an integral role in body weight regulation [122], although the precise mechanism(s) by which it mediates this effect is currently debated [123]. Expectedly, leptin levels track with body fat changes in mice chronically exposed to diets of varying macronutrient compositions [58,60], with no linear relationship between dietary protein and leptin owing to an inverted U shaped curve effect on fatness mediated by dietary protein intake [58]. Leptin seems to be permissive for the effects of protein restriction on systemic metabolic inefficiency [124], and affects the improved stress-resistance of the kidney to ischemia-reperfusion injury [125]. Clearly further research is warranted on the role and regulation of leptin and leptin signalling in the effects of acute and chronic effects of altered dietary protein intake.

### 5.2. Adiponectin

Adiponectin is secreted from adipose tissue in a fashion inversely related to fatness [126] and regulates whole body energy homeostasis by mainly acting on the brain and liver [126]. In mice, chronic dietary protein restriction increases adiponectin along with reduced fatness [60]. While there is sparse information relating to the role of this change, a recent study demonstrated that reduced adiponectin may play a role in modulating female fertility in response to chronic dietary protein restriction [110,127].

## 6. Summary and Future Directions

**Figure 1:**
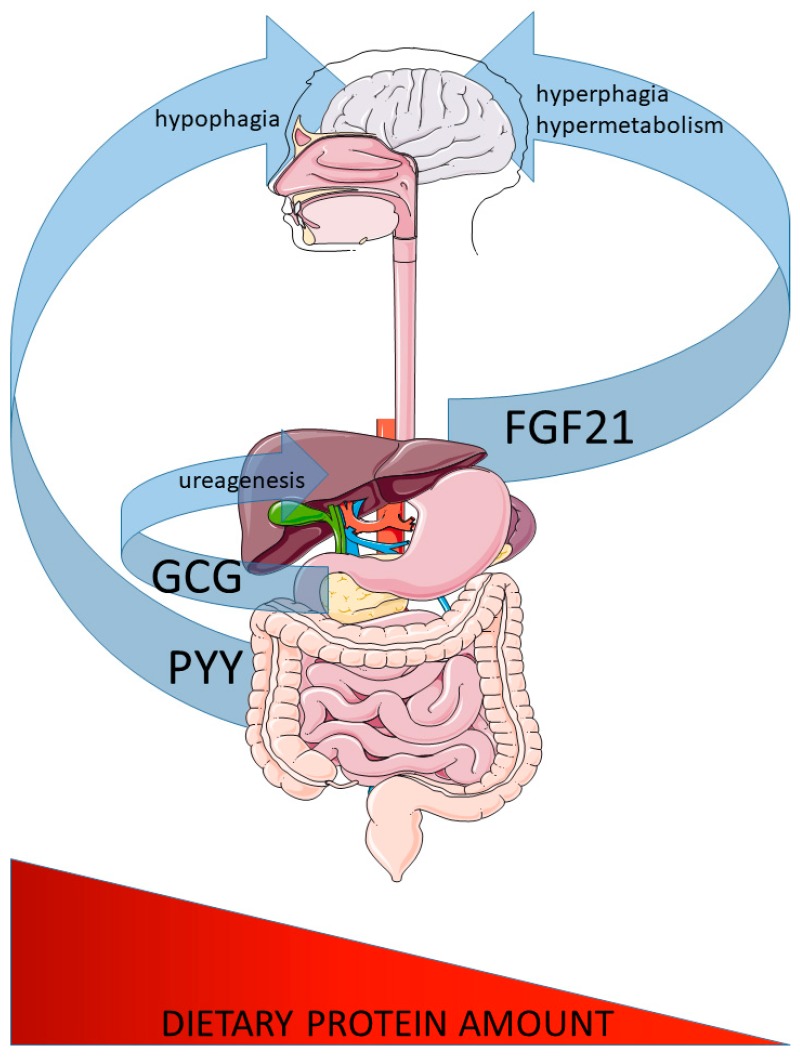
Major peptide hormones controlling systemic responses to altered dietary protein intake. Glucagon (GCG) and peptide-tyrosine-tyrosine (PYY) secretion are increased from the pancreatic islet alpha cells (GCG) and intestine (PYY) in response to increased protein intake. GCG affects liver ureagenesis to defend systemic amino acid homeostasis and PYY confers the increased satiation by acting on brain feeding regulatory circuits. In contrast, fibroblast growth factor 21 (FGF21) is secreted from the liver in response to low dietary protein intake and confers increased feeding and systemic metabolism via brain action.

Aside from several intracellular signalling cascades, peptide hormones play a major role in mediating the systemic metabolic response to alterations of dietary protein intake. In particular, both glucagon and PYY are mediators of the response to heightened protein intake modulating liver amino acid metabolism and hypophagia, respectively, while FGF21 has emerged as a bone-fide endocrine signal of low protein intake affecting both the altered feeding and systemic metabolic response (Figure 1). Future studies will certainly uncover more roles of these, and perhaps novel, peptide hormones and their roles in how dietary protein intake affects mood, eating behaviour, reproduction, cardiovascular responses, and brain health. A particular focus should be the adoption of the geometric framework for nutrition, as the one-nutrient-at-a-time approach often does not simulate real-world situations and complex nutrient interactions can be overlooked [128]. Moreover, further efforts should be made to distinguish between acute and chronic effects of dietary protein dilution/enrichment, as well as sex- and age-dependent nutrient-hormone interactions. Finally, with the adoption of new technologies and fields of study such as high-throughput proteomics and metabolomics as well as exosomes as non-canonical hormone carriers, novel systemic signalling modulators will undoubtedly be revealed that link alterations in organismal homeostatic responses to the intake of the critical nutrient protein.
